# Milder is better? advantages and disadvantages of "mild" ovarian stimulation for human in vitro fertilization

**DOI:** 10.1186/1477-7827-9-25

**Published:** 2011-02-16

**Authors:** Alberto Revelli, Simona Casano, Francesca Salvagno, Luisa Delle Piane

**Affiliations:** 1 Reproductive Medicine and IVF Unit, Department of Obstetrical and Gynecological Sciences, University of Torino, via Ventimiglia 3, 10126 Torino, Italy

## Abstract

In the last decades, several steps have been made aiming at rendering human IVF more successful on one side, more tolerable on the other side. The "mild" ovarian stimulation approach, in which a lower-than-average dose of exogenous gonadotropins is given and gonadotropin treatment is started from day 2 to 7 of the cycle, represents a significant step toward a more patient's friendly IVF. However, a clear view of its virtues and defects is still lacking, because only a few prospective randomized trials comparing "mild" vs. conventional stimulation exist, and they do not consider some important aspects, such as, e.g., thawing cycles. This review gives a complete panorama of the "mild" stimulation philosophy, showing its advantages vs. conventional ovarian stimulation, but also discussing its disadvantages. Both patients with a normal ovarian responsiveness to exogenous gonadotropins and women with a poor ovarian reserve are considered. Overall, we conclude that the level of evidence supporting the use of "mild" stimulation protocols is still rather poor, and further, properly powered prospective studies about "mild" treatment regimens are required.

## Background

Since the early ages of human in-vitro fertilization (IVF) it turned out clearly that the effectiveness of the procedure when performed on a natural, single-egg cycle was very limited. An important step toward getting better results was represented by the availability of medications able to induce multiple ovulation. For several years, and until now, ovarian stimulation with exogenous hormones has been widely applied with the aim of increasing the number of oocytes available for fertilization [1]. For years pharmaceutical companies have been competing on the market using as a tool the potency of their respective drugs to get more oocytes. Cancelling cycles in which ovarian stimulation obtains a low number of developing follicles has become a popular choice, especially in Countries in which the fierce competition among IVF clinics is based on the pregnancy rate, and thus it is inconvenient to go on with cycles in which a poor oocyte yield is predictable. Furthermore, in Countries where either the public health system or the private insurance system offer for free (or at very low costs) only a limited number of attempts, the yield of at least a dozen of oocytes is considered of great value by IVF doctors and, as a consequence, by patients. Again, IVF clinics running an oocyte donation program are particularly satisfied when a patient produces enough eggs to be treated herself and to give surplus oocytes to donation. More oocytes-more embryos-more pregnancies = better IVF program: the most widely accepted principle all over the IVF world.

However, it is out of discussion that the need of getting a rather high number of oocytes arises from the overall inefficiency of IVF laboratory procedure: several oocytes are needed to finally get just a few embryos and much less born babies. It is easy to calculate that the live birth rate/inseminated oocyte is extremely low in human IVF, on the average around 2-4%. Thus, the complex and demanding ovarian stimulation protocols are usually applied in order to compensate for the poor laboratory effectiveness.

The IVF lab has indeed improved significantly in the past three decades: new media and new equipment for embryo culture have been made available on the market, new scientific knowledge has been obtained. As a result, the overall efficiency of IVF procedure has markedly improved from the 80's until now: is it still necessary to work on a high number of oocytes to get a baby?

## Recruitment, selection and dominance of follicles in ovarian physiology

The complete follicular development in humans takes about 220 days and includes three distinct phases according to the developmental stage and to the dependence from pituitary gonadotropins: (a) initial recruitment of resting primordial follicles, (b) development of preantral and early antral follicles, (c) cyclic recruitment of a limited cohort of antral follicles followed by the selection of a single dominant follicle [2]. Phases (a) and (b) are regulated by a complex interplay of intra-ovarian factors and are independent on gonadotropins. Phase (c) begins during the luteal-follicular transition of the menstrual cycle, when follicle-stimulating hormone (FSH) circulating levels rise and increase over a threshold at which a cohort of small antral follicles is recruited to grow [3].

The beginning of follicular growth is characterized by morphological changes, including the proliferation of granulosa cells, their change in shape, the enlargement of the oocyte and the formation of the zona pellucida. The early theca is acquired at the end of the primary follicle stage, whereas the external theca forms as the follicle grows and compresses the surrounding stroma [3]. During the early preantral follicle development, FSH, estrogen and androgen receptors appear on the granulosa cell surface; however, at this stage, the follicle is still unaffected by the lack of gonadotropins [2]. After the initial rise, FSH blood levels plateau during the early follicular phase and finally decrease during the mid-to-late follicular phase as a consequence of the negative feedback exerted by inhibin B and estrogens on the hypothalamic-pituitary axis [2].

The presence of FSH is an absolute requirement for the development of larger antral follicles [2]. Around the mid-follicular phase, the most mature follicle (the one with the highest number of FSH receptors on granulosa cells) gains dominance over the others; despite progressively decreasing FSH blood levels, the dominant follicle continues to grow and acquires responsiveness to LH. The remaining follicles from the recruited cohort undergo atresia and programmed cell death [1]. The time during which FSH blood concentration is above a given threshold appears to be essential for a single dominant follicle selection (*FSH window*) [1].

The theorical concept at the basis of the "mild" stimulation strategy is that a moderate but continued elevation of FSH during the mid-to-late follicular phase is able to extend the FSH window and to overcome the single dominant follicle selection, leading to the growth of several follicles [2]. In the "mild" ovarian stimulation, a low-dose gonadotropin administration is delayed until the mid-follicular phase (day 2-to-5 of the cycle). The aim is to allow initial follicle recruitment by endogenous FSH, then prevent the decrease of FSH levels overcoming dominance and inducing multi-follicular development [1]. In women with a normal ovarian follicular reserve, multiple follicle development can be induced when the initiation of FSH is postponed until cycle day 7, although there is a tendency toward a higher percentage of monofollicular responses compared with patients starting on cycle day 2-to-5. A fixed daily dose of 150 IU FSH is usually enough to induce multiple follicular growth when ovarian stimulation is initiated on cycle day 5 [4].

## Conventional "long"protocols and the "mild" stimulation approach

In the conventional "long" protocol, gonadotropins (FSH, hMG or FSH+LH) are given to induce multiple follicular development and a GnRH analogue is contemporaneously given to prevent the premature LH surge, that would compromise the chance of retrieving oocytes [5]. It took approximately 15 years of experience with GnRH agonists to identify the optimal way to use them; the best results were obtained starting GnRH analogue administration in the mid-luteal phase of preceding cycle, the so-called "long" protocol [6,7].

In the "long" protocol, GnRH agonist is started in the luteal phase of the run-in cycle and continued until the administration of hCG (ovulation trigger). An initial flare of gonadotropin release (about 5 days) takes place before the receptors are down-regulated and GnRH action on the pituitary is blocked [6]. This protocol, probably the most widely used throughout the world even now, allows a quite good predictability of the work in IVF Units, implies a low cancellation rate, and allows to get a relatively high number of pre-ovulatory follicles, of retrieved oocytes and, as a consequence, of embryos available for transfer, thus leading to a satisfactory pregnancy rate [8].

However, the "long" protocol is associated with some problems: several risks and complications have been reported, the most important being ovarian hyperstimulation syndrome (OHSS) in its severe form [9]. Hundreds of patients are hospitalized every year because of severe OHSS, which represents a life-threatening, high-cost complication of human IVF. Moreover, the complexity of the "long" protocol, entailing weeks of daily injections and/or intra-nasal spraying, several blood samples and frequent ovarian ultrasound scans for monitoring, can impact on a woman' s life, causing a high rate of drop-out from IVF program [8].

The "mild" stimulation approach for IVF treatment is aimed to develop more patient-friendly protocols in which the risks are minimized and the results are still acceptable [8]. A "mild" IVF cycle is defined (ISMAAR association) either as (a) a stimulation regimen in which gonadotropins are administered at a lower-than-usual dose and/or for a shorter duration throughout a cycle in which GnRH antagonist is given as co-treatment, or (b) a stimulation in which oral compounds (e.g. anti-estrogens) are used either alone or in combination with gonadotropins and GnRH-antagonists [10].

## The key role of GnRH antagonists in "mild" stimulation regimens

Although the use of GnRH antagonists is probably not absolutely required for "mild" ovarian stimulation [11], their introduction in the clinical practice has represented the key event to start using "mild" protocols in IVF. The action of GnRH antagonists is characterized by an immediate suppression of the pituitary release of gonadotropins and by a rapid recovery of normal gonadotropin secretion when the drug is withdrawn [6]. The mid-cycle LH surge requires the secretion of native GnRH and can therefore be effectively prevented by GnRH antagonist administration. The immediacy of action of GnRH antagonists allows to block the pituitary just when the circulating estradiol rise approaches the threshold level at which LH surge is generated by the positive feedback loop on the pituitary. At the beginning of stimulation cycle; ovarian stimulation can be initiated by endogenous gonadotropins with a normal early follicular phase recruitment of a cohort of follicles, without any pituitary block [6].

In the last years, three general approaches for the GnRH antagonist co-treatment in IVF have emerged: (a) inject a single large dose subcutaneously on approximately the eighth day of stimulation with gonadotropins, (b) give daily injections of small doses initiated on a fixed day of stimulation (*fixed protocol*), or (c) give daily injections of small doses initiated depending on the size of the dominant follicle or on estradiol levels (*flexible protocol*) [6]. Administration regimens (b) and (c) are continued until the day in which hCG is given to obtain the final oocyte maturation.

The introduction of GnRH-antagonists in the clinical IVF workout has been characterized by a long learning curve (probably still ongoing) and by several perplexities on their effectiveness. Initially, large prospective trials comparing ovarian stimulation with the "long" agonist protocol vs. a daily antagonist protocol (in which high gonadotropin doses were used) revealed the latter to generate fewer follicles and fewer oocytes; oocytes and embryos were of equivalent morphological quality, but finally an average decrement in pregnancy rate of 5% was observed in the antagonist cycles [12]. The better pregnancy rate in the agonist cycles was felt to be due to the larger oocyte availability, allowing to obtain more embryos and to better select those worth to be transferred in utero or cryopreserved [13]. Moreover, in the initial dose-finding studies on GnRH antagonists an inverse relationship between antagonist dose and implantation rate was observed, suggesting a potential toxic effect of the drug on embryos and/or on the endometrial receptivity [13]. After these early reports were published, the protocols with GnRH antagonists became for many clinicians a second choice; this is proven by the analysis of the German IVF registry from 2000 to 2003, that clearly shows that GnRH antagonists were often utilized in cycles with unfavourable *a priori *prognosis (e.g. patients with advanced reproductive age and/or with several previously failed IVF attempts) [14]. GnRH antagonists began to be revaluated when the sub-analysis of patients with equal demographic and clinical features was performed on the same database: similar pregnancy rates were found independent of whether GnRH agonist or antagonist had been used [15]. Further, although the initial five large randomized controlled trials (RCTs) comparing GnRH antagonists vs. agonists showed a 5% difference in the chance of clinical pregnancy in favour of the agonists, the difference in live birth rate (still in favour of agonists by 3.8%) was not statistically significant [16]. A later meta-analysis including 22 RCTs published between 2000 and 2005 and including much more patients (3176 women) found a live birth rate reduced by -2.7% in the antagonist-treated group, still not statistically significant even when data were subdivided and re-analysed according to the type of population, the type of gonadotropin used, the type of agonist and antagonist [16]. Differently, the Cochrane Review including 27 RCTs (3865 patients), observed significantly lower clinical and ongoing pregnancy rates in women treated with antagonists (OR 0.84, 95% CI = 0.72-0.97 and OR 0.82, 95 CI 0.69-0.98 respectively) [17], but still these results could be interpreted as due to the learning curve needed to optimize protocols with antagonists, similarly to what happened when agonists were made available on the market.

## IVF results in "mild" stimulation protocols vs. classical "long" protocol

At present, the "long" GnRH-agonist regimen with relative high doses of exogenous gonadotropins is probably the most frequently used stimulation protocol; this regimen has become a comfortable routine over the years in many IVF clinics. In the last years, the availability of GnRH antagonists has allowed the clinical development of "mild" ovarian stimulation protocols involving subtle interference with single dominant follicle selection. Some studies have compared the success rate of "mild" vs. standard ovarian stimulation regimens, either in women with normal ovarian reserve or in subjects with poor ovarian reserve.

### Women with normal ovarian reserve

Three prospective, randomized controlled trials compared the effectiveness of "mild" stimulation regimen with the conventional "long" GnRH agonist protocol with an early follicular phase FSH start.

The single centre RCT by Hohmann et al. [18] included 142 patients with good reproductive prognosis who were randomly distributed into three groups: (A) (n= 45) treated with the GnRH-agonist Triptorelin and, after down-regulation, with a fixed daily dose of 150 IU rFSH; (B) (n= 48) and (C) (n= 49) treated with rFSH initiated on cycle day 2 (group B) or 5 (group C) and with the GnRH-antagonist Cetrorelix starting when the largest follicle reached 14 mm diameter. Group C showed a shorter duration of stimulation, reflected in a significantly lower total dose of exogenous rFSH; in group C, more cycles were cancelled due to insufficient response and less oocytes were harvested, but better quality embryos were finally obtained. Fewer cycles in group C were characterized by a total fertilization failure or by abnormal embryo development. Nevertheless, because of the higher number of oocytes retrieved in group A and B and to the better availability of embryos among which selecting the best, no significant difference in the quality of the best transferred embryo was observed among the three groups. After stronger ovarian stimulation (groups A and B), only 7% of the patients who retrieved less than 5 oocytes conceived, whereas after "mild" stimulation 67% of these patients conceived. Overall, no differences were found among the three groups as far as the pregnancy rate per started cycle was concerned.

The results of this study suggest that a low number of retrieved oocytes after "mild" ovarian stimulation could have a different meaning from a low number of retrieved oocytes after a conventional stimulation. It was shown that the ideal number of oocytes after a conventional "long" protocol is 13 and that when the number of retrieved eggs is much lower (or even much higher), the pregnancy rate is compromised [19]. A poor oocyte yield after classical ovarian stimulation likely reflects a poor ovarian responsiveness to FSH, that is associated with poor IVF outcome, whereas a low number of oocytes after "mild" stimulation probably represents a normal response, a smoother selection of follicles (and oocytes) more likely to finally result in high quality embryos and in a pregnancy [20]. A low overall dose of exogenous FSH probably stimulates only the most mature follicles having optimal receptor endowment, and allows a sort of "quality selection" among follicles (and finally among oocytes) avoiding to force poor quality follicles to grow anyway [20].

Another RCT by Heijnen et al. [21] included 404 regularly cycling, normal BMI patients and almost 800 consecutive IVF cycles. In this study, one group was given "mild" ovarian stimulation with GnRH antagonist co-treatment combined with selected single embryo-transfer (SSET), whereas the other received a standard ovarian stimulation with the GnRH agonist "long" protocol combined with the transfer of two embryos. The "mild" treatment group was characterized by a lower duration of stimulation, a lower total FSH dose and by less retrieved oocytes. The pregnancy rate per cycle was significantly lower in the "mild" stimulation group (17.6% vs 28.6%, p < 0.0001); however, the "mild" stimulation protocol, easier to stand and cheaper for patients, reduced the rate of treatment discontinuation and induced some patients to undergo shortly a second IVF attempt. This attitude resulted in a cumulative live birth rate after 1 year of IVF treatments that was comparable in the two groups (43.4% with the mild protocol, 44.7% with the standard regimen), and the twinning rate was significantly lower in the "mild" stimulation-SSET transfer group (0.5% vs. 13.1%, p < 0.0001) [21]. According to this study, a reduced chances of birth per cycle in the "mild" regimen might be compensated by the increased number of IVF attempts in a set time. In fact, the overall discomfort to patients, evaluated with the hospital anxiety scale, the depression scale, the somatic Hopkins' subscale and the subjective sleep quality scale, were lower in the group assigned to the "mild" stimulation. Interestingly, also the economical costs of the treatment were significantly lower with the mild stimulation/SSET compared with a standard treatment involving conventional stimulation; it must be remarked, however, that the money saving was mainly due to the almost complete absence of twin pregnancies in the "mild" group rather then to the cost of IVF procedure itself [21]. Interestingly, a prediction model aimed to estimate the chance of ongoing pregnancy after "mild" stimulation and SSET has been prepared using multivariate logistic regression analysis; this model is claimed to be helpful to optimize results identifying patients for which the "mild" stimulation plus SSET strategy can be appropriate [22].

The third RCT comparing the "mild" protocol and the "long" classical protocol was performed by Baart et al [23] on 111 patients who were randomized into two groups, one (n = 67) receiving rFSH 150 IU/day from day five of the cycle plus GnRH-antagonist in a flexible schedule, the other (n = 44) receiving rFSH 225 IU/die after two weeks of pituitary down-regulation by GnRH-agonist. In this study, couples with severe male factor were excluded. The ongoing pregnancy rate per started cycle was 21% in the "mild" group and 18% in the control group, a difference not statistically significant. Interestingly, in this study embryos were biopsied at the 8-cells stage and 1 or 2 blastomeres were then analyzed by fluorescent in-situ hybridization (FISH) considering 10 chromosomes: despite "mild" stimulation obtained significantly fewer oocytes and embryos, both regimens finally generated the same number (1.8/cycle) of chromosomically normal embryos. This observation suggests that the reduced pharmacological interference with ovarian physiology could generate oocytes of better genetical quality. Indeed, some data in the mouse model show that exogenous disturbances in the signals regulating folliculogenesis may alter the late stage of oocyte growth, increasing the risk of altered chromosome segregation in subsequent meiotic divisions: an increased incidence of chromosomal abnormalities, in fact, was observed in oocytes after exposure to high doses of gonadotropins during in vitro maturation of mouse oocytes [23,24]. Mild stimulation approaches, aiming at a more physiological response, might be able to improve the genetical quality of oocyte and embryos in humans. This hypothesis, however, needs to be validated by further trials including a higher number of patients and embryos, and possibly using techniques (e.g. CGH) able to study the whole set of chromosomes on a single blastomere.

Taken together, these three RCTs comparing the "mild" with the classical stimulation regimen included 592 first IVF attempts, among which 313 were performed with the "mild" stimulation protocol and 279 with the classical "long" regimen. Although individually these trials found comparable results in terms of IVF effectiveness, pooling data together the ongoing pregnancy rate per started cycle sorts out to be 15% in the "mild" group and 29% in the classical group, a difference that suggests a well definite trend toward a lower IVF effectiveness when the "mild" strategy is applied. This suggestion is further reinforced by the fact that the three RCTs did not consider freeze-thaw cycles, and the chance of obtaining surplus embryos/oocytes to freeze is obviously much lower in "mildly" stimulated cycles than in the classical ovarian stimulation cycles. Having frozen embryos/oocytes to transfer in a subsequent cycle can probably increase the overall IVF pregnancy chance per oocyte pick-up by approximately 10-15%: thus, the gap between the two competitors could probably be wider considering freeze/thawing cycles, and could possibly reach statistical significance. New RCTs including freeze-thaw cycles in the comparison between "mild" and classical stimulation regimens are definitely needed to get a higher level of evidence about the respective effectiveness of the two strategies.

A factor limiting the effectiveness of "mild" strategy in terms of pregnancy rate per cycle is likely to be the relatively high rate of cycle cancellation due to mono- or bi-follicular response (around 15-20%) when gonadotropins are started on day 5 of the cycle. When such a response is observed, a valuable option is to stop stimulation and start it again the next month starting earlier with medications (on day 2-4). Ovarian aging and high BMI have been identified as relevant variables to predict the risk of insufficient response to "mild" stimulation, and a predictive model have been developed in order to minimize the need of cancelling the cycle [25].

On the other side, classical ovarian stimulation is claimed to be a factor able to impair endometrial maturation and consequently embryo implantation chance. Some studies indeed showed that the gene expression profiling of the endometrium in conventionally stimulated cycles is extremely different from the one that can be observed during a natural cycle [26,27]: interestingly, the endometrial gene expression pattern is more similar to the natural one in cycles with GnRH-antagonists than in cycles with GnRH-agonists [28]. Furthermore, classical ovarian stimulation regimens are associated with about ten-folds supra-physiological circulating estradiol levels that have a well documented negative impact on the developmental and implantation potential of human embryos [24,29]. Some data suggest that the best endometrial receptivity to embryo implantation may be found in natural cycle, but mild stimulations have a lower impact on endometrial quality than classical regimens [30,31]. The "mild" stimulation regimen is associated with significantly lower peak estradiol levels, and possibly can impact on the endometriom more softly than classical regimens: the "endometrial factor" can probably represent one scored point in favour of "mild" stimulation.

### Women with poor ovarian reserve

Ovarian stimulation for women with a poor ovarian reserve is probably one of the most frustrating aspects of IVF procedure: most of the treatments proposed to enhance oocyte yield and pregnancy rates in "poor" responders have failed to show any convincing evidence [32].

The standard approach to women estimated to be poor responders is based on starting the "long" protocol with a daily dose of approximately 300 FSH IU/die [33]; the starting FSH dose used in any subsequent cycle is then adjusted (up to 600 FSH IU/die) according to the individual response in the first cycle [34]. The strategy of performing an upward dose adjustment in women with poor ovarian reserve, however, has not shown any consistent benefit. In previous poor responders, in fact, the IVF outcome of those assigned to a starting dose of 225 FSH UI/day vs. those receiving 450 UI/day was shown to be similar, despite the latter obtained more oocytes [35]. Also other studies showed that predicted poor responders had no benefit from increasing the FSH starting dose [36,37]. High gonadotropin doses may indeed lower the cycle cancellation rate, but have been observed to reduce the likelihood of clinical pregnancy and live birth rate and to increase the risk of spontaneous miscarriage [38]. A negative effect of high dose regimens on the endometrial quality has been claimed to be responsible for the poor outcome of this regimen [39], although probably even factors linked to embryo quality itself play a relevant role. High doses of FSH recruit "resistant" follicles rescuing them from atresia, but the oocytes that they host are of poor quality and usually do not result in the generation of good quality embryos [40,41]. Since the economical cost of gonadotropins is one of the major expenditures in IVF treatment, the huge increase in drug cost linked to high-dose gonadotropin regimens appears to be a nonsense if not paralleled by a significant improvement in clinical outcome.

A combination of Clomiphene citrate (CC) plus gonadotropins and GnRH antagonists has been proposed as a "mild" stimulation alternative for poor responders. CC is known to act as an anti- estrogen on the central nervous system, increasing the pulse frequency of endogenous FSH and LH and giving a moderate gonadotropin stimulus to the ovary [42]. CC has been used for over thirty-five years to induce ovulation in WHO type II anovulatory women, and is still appreciated for its oral administration and low price; the combination with gonadotropins may counterbalance its undesired anti-estrogenic effect on the endometrium and at the same time may reduce the amount of gonadotropins required, thanks to the combined synergistic effect on the ovary.

The level of evidence supporting the use of the "mild" stimulation protocol with CC/Gn/GnRH antagonist in patients with poor ovarian reserve is rather poor, as properly designed studies on an appropriate number of patients are still unavailable. The first report describing the use of CC/Gn/GnRH antagonists in poor responders included only eighteen patients; compared to their response in a previous standard GnRH-agonist cycle, light improvements in cycle cancellation rate, oocyte yield and gonadotropin requirement were observed [43]. Unfortunately, neither the number of patients, nor the study design allowed to get an acceptable level of evidence.

Takahashi et al. [44] studied 40 poor responders with a story of multiple IVF failures with the "long" protocol: treating them with CC/FSH/GnRH antagonist he obtained an ovarian response comparable to the previous ones, but a significantly higher blastocyst development rate and a very good (41.2%) ongoing pregnancy rate. Again, the study design was not very informative and the patient number was too little.

D'Amato et al [45] compared the combination of CC/FSH/GnRH antagonist to a long GnRH agonist protocol enrolling 145 women with a prior poor response; a significantly lower cancellation rate, higher peak oestradiol level, more retrieved oocytes and higher pregnancy and implantation rates were achieved with the antagonist protocol. In this study, however, high FSH amounts and not a "mild" gonadotropin stimulation were used: the observations, once again, are only indicative of the possibility of using this kind of stimulation at lower doses in poor responders.

Some other informations may be deduced from studies that compared the outcome of CC/Gn/GnRH antagonist treatment with a standard "long" protocol in patients with normal ovarian reserve: pregnancy rates comparable to the standard stimulation regimens were obtained by the "mild" strategy, with a significant reduction in the total dose of gonadotropin needed and of the economical costs [46-48]. These results appear to be encouraging, although it remains to be proven that they can be replicated even with patients with poor ovarian reserve.

Interestingly, it was shown that when in CC/Gn/GnRH antagonist cycles the circulating level of LH is less than one-third at the time of hCG than it was at the beginning of stimulation, both the pregnancy and implantation rates are significantly reduced [49]: this observation suggests to chose medications containing LH or hCG rather than FSH alone to be associated with CC in this kind of protocol.

Overall, the published results suggest that in patients with poor ovarian reserve the choice of a mild stimulation protocol instead of a classical, high dose regimen, could be particularly indicated. Although these patients have a very low risk of OHSS even using high doses, the quality of both their oocytes and their endometrium could likely to be better when a smoother stimulation approach is used. Further research, anyway, is needed to add scientific evidence to this hypothesis.

## Other aspects to be considered

### Risk of ovarian hyperstimulation syndrome (OHSS)

It is well known that IVF treatment exposes women to the risk of short-term complications, among which the severe form of OHSS is the most important. Severe OHSS is a serious and potentially life-threatening complication of IVF whose symptoms are very well known, and has a mean incidence of 1-3% in IVF programs involving standard ovarian stimulation regimens [50]. Some patients are definitely considerable at high risk: young, lean women with polycystic ovaries (PCO) are a typical example; their risk of developing severe OHSS after a conventional ovarian stimulation for IVF is around 6-9% for a young PCO patient.

The incidence of severe OHSS is significantly lower when GnRH antagonists are used instead of agonists [16,17], probably due to the smaller cohort of recruited follicles and to the lower circulating estradiol levels during ovarian stimulation. The meta-analysis of Kolibianakis found that the incidence of hospital admission for OHSS is significantly lower in GnRH antagonist cycles than in agonist cycles (OR 0.46;95% CL 0.26-0.82; P = 0.1) [16]. Also the Cochrane review by Al-Inany reported that the incidence of severe OHSS is significantly lower in protocols with GnRH antagonists than in protocols with GnRH agonists (RR 0.61; 95% CI 0.42-0.89; P = 0.01); moreover, secondary methods to prevent OHSS (such as coasting or cycle cancellation) need to be used more frequently during the GnRH-agonist cycles (OR 0.44;95% CI 0.21-0.93;P 0.03) [17]. Further, the risk of severe OHSS is reduced until around zero if ovulation trigger is elicited using a single dose of GnRH agonist instead on hCG: this is possible if a stimulation with GnRH antagonists has been applied, and an endogenous LH peak can be obtained stimulating the pituitary [51].

The risk of developing severe OHSS is further significantly reduced using "mild" stimulation regimens; in the study of Heijnen [21] the incidence of OHSS was 1.4% with the mild protocol and 3.7% with the long protocol. In another study, Karimzadeh et al. [46] observed a zero incidence of OHSS in the group treated with "mild" stimulation vs. 6% in the group treated with conventional stimulation.

### Risk of long term health problems

The discussion about long-term health consequences of ovarian stimulation for IVF, especially concerning the association between hormones and cancer, is far from being concluded. The epidemiological studies published so far in humans remain inconclusive, due to a huge amount of confounders and to a relatively short follow-up period of time [52,53].

Although gonadotropin treatment is not considered oncogenic, nor able to significantly affect the patient's chance of having serious diseases, it appears safer to use the lowest dose possible of hormons, expecially in case of repeated IVF attempts.

### Emotional stress

Emotional stress represents a well known negative side effects associated with IVF treatment, and probably one of the most important reasons for dropping out of the program.

Some studies suggest that women who receive milder approaches in ovarian stimulation could be more prone to face a new treatment attempt compared with women receiving a standard protocol: in fact, the psychological burden of treatment is one of the most frequent causes of drop-out, and a significantly lower drop-out rate was observed in more patient-friendly "mild" stimulation programs [22,54]. The lower incidence of the so-called minor symptoms (abdominal pain, nausea, etc.) that can be associated with conventional ovarian stimulation protocols is probably a factor that increases the patients' attitude to repeat IVF, as it has a lower daily impact on the quality of life. A better predisposition to repeat the treatment after a failed attempt may obviously have a positive impact on the cumulative treatment success rate and could eventually compensate for a lower pregnancy rate per cycle following "mild" stimulation.

On the other side, however, the lower pregnancy chance per attempt with the "mild" approach can be itself a reason of emotional stress. Treatment failure, in fact, is associated with a deterioration of emotional well being [55], subclinical depression [56] and/or anxiety [57]. Furthermore, one of the most stressing events in an IVF cycle is oocyte retrieval, either preformed under general or local anaesthesia; if a stimulation strategy gets lower "per cycle" results, patients will be more frequently forced to repeat oocyte retrieval, with a possible increase of emotional stress.

Some studies comparing the conventional ovarian stimulation with the "mild" regimen failed to observe a difference in the anxiety or depression levels, or on sleep quality [21,58].

Overall, the evidence about a possible psychological benefit of "mild" protocols is still inconclusive. Even when looking at the patient's emotional well-being, a careful balance between the daily physical problems of stimulation and the overall effectiveness of IVF treatment must be carefully considered, discussing with each single couple pro and contra of the choice of a specific protocol.

### Economical costs

A milder ovarian stimulation is undoubtedly associated with a lower medication consumption and with a lower cost for purchasing drugs. A couple of studies showed the superiority of the "mild" stimulation strategy over the standard approach in terms of economical costs [21,59], especially when the "mild" strategy includes SSET [21].

Once again, however, the balance between lower costs and lower "per cycle" results must be carefully considered: if IVF effectiveness is under a critical threshold, the frequent need to repeat treatment (even several times) leads to new economical costs for the patient. This could represent a concrete problem particularly for people living in those countries where patients pay for their own treatment or the public health/private insurance systems give for free (or at very low costs) only a limited number of attempts. The convenience of adopting the "mild" stimulation strategy in developing "third world" Countries has not yet been convincingly demonstrated.

The lower effectiveness of IVF procedure can also become a problem for IVF clinics choosing the "mild" strategy, who will compete on the market with clinics following classical stimulation concepts; if the clinic loses patients for the lower "per cycle" effectiveness of its IVF program, it could be forced to go back to classical stimulations or, alternatively, to increase prices, finally weighting on patients' budget.

## Conclusions

In the last decades, several steps have been done with the aim of rendering human IVF more and more successful on one side, more and more tolerable and easy to perform on the other side. IVF programs are now totally feasible on an outpatient, office basis: no hospitalization or general anaesthesia are needed, and patients more or less keep on with their normal life during treatment. Anyway, a considerable proportion of women still experience discomfort and emotional stress, and some of them face relevant (or even serious) health complications. Moreover, IVF is still a very expensive treatment, especially considering its limited success rate that often forces to repeated attempts and the higher chance of having a twin pregnancy with all its obstetrical and neonatal complications.

The mild stimulation approach, especially when linked to selected single embryo transfer, may represent an important step toward the objective of an easier IVF, more tolerable and problem-free for patients, cheaper for both patients and the society, but still having an acceptable effectiveness in terms of baby birth rates.

Effectiveness is actually the core of discussion when dealing with "mild" stimulation strategy. To date, too few properly designed studies are available to allow a scientific, conclusive judgement. What is definitely needed is a series of RCTs comparing, in different subsets of IVF patients, "mild" stimulation protocols with the conventional GnRH agonist protocols or, alternatively, with stimulation regimens using GnRH antagonists with high gonadotropin doses. To be fully informative, these studies should come from different research groups and should be properly weighted and designed, involving also freeze-thaw cycles.

The need for an OHSS-free IVF program is felt as a key issue by most IVF doctors and represents one of the objectives of many IVF Units. Further research is needed to find ideal ovarian stimulation protocols able to obtain high quality oocytes, optimal IVF success rates even in poor-prognosis patients and in the meanwhile able to reduce undesirable effects and complications. The "mild" stimulation philosophy is interesting, but its true effectiveness as well as its impact on emotional and economical aspects of IVF must be further investigated.

## Competing interests

The authors declare that they have no competing interests.

## Authors' contributions

All authors contributed to bibliographic research, critical evaluation of the articles included in the manuscript and writing of the manuscript. All authors read and approved the final manuscript.
